# From Spiking Neuron Models to Linear-Nonlinear Models

**DOI:** 10.1371/journal.pcbi.1001056

**Published:** 2011-01-20

**Authors:** Srdjan Ostojic, Nicolas Brunel

**Affiliations:** 1 Center for Theoretical Neuroscience, Columbia University, New York, New York, United States of America; 2 Laboratoire de Physique Statistique, CNRS, Université Pierre et Marie Curie, Université Paris-Diderot, Ecole Normale Supérieure, Paris, France; 3 Laboratory of Neurophysics and Physiology, CNRS UMR 8119, Université Paris Descartes, Paris, France; Gatsby Computational Neuroscience Unit, UCL, United Kingdom

## Abstract

Neurons transform time-varying inputs into action potentials emitted stochastically at a time dependent rate. The mapping from current input to output firing rate is often represented with the help of phenomenological models such as the linear-nonlinear (LN) cascade, in which the output firing rate is estimated by applying to the input successively a linear temporal filter and a static non-linear transformation. These simplified models leave out the biophysical details of action potential generation. It is not a priori clear to which extent the input-output mapping of biophysically more realistic, spiking neuron models can be reduced to a simple linear-nonlinear cascade. Here we investigate this question for the leaky integrate-and-fire (LIF), exponential integrate-and-fire (EIF) and conductance-based Wang-Buzsáki models in presence of background synaptic activity. We exploit available analytic results for these models to determine the corresponding linear filter and static non-linearity in a parameter-free form. We show that the obtained functions are identical to the linear filter and static non-linearity determined using standard reverse correlation analysis. We then quantitatively compare the output of the corresponding linear-nonlinear cascade with numerical simulations of spiking neurons, systematically varying the parameters of input signal and background noise. We find that the LN cascade provides accurate estimates of the firing rates of spiking neurons in most of parameter space. For the EIF and Wang-Buzsáki models, we show that the LN cascade can be reduced to a firing rate model, the timescale of which we determine analytically. Finally we introduce an *adaptive timescale rate model* in which the timescale of the linear filter depends on the instantaneous firing rate. This model leads to highly accurate estimates of instantaneous firing rates.

## Author Summary

Deciphering the encoding of information in the brain implies understanding how individual neurons emit action potentials (APs) in response to time-varying stimuli. This task is made difficult by two facts: (i) although the biophysics of AP generation are well understood, the dynamics of the membrane potential in response to a time-varying input are highly complex; (ii) the firing of APs in response to a given stimulus is inherently stochastic as only a fraction of the inputs to a neuron are directly controlled by the stimulus, the remaining being due to the fluctuating activity of the surrounding network. As a result, the input-output transform of individual neurons is often represented with the help of simplified phenomenological models that do not take into account the biophysical details. In this study, we directly relate a class of such phenomenological models, the so called linear-nonlinear models, with more biophysically detailed spiking neuron models. We provide a quantitative mapping between the two classes of models, and show that the linear-nonlinear models provide a good approximation of the input-output transform of spiking neurons, as long as the fluctuating inputs from the surrounding network are not exceedingly weak.

## Introduction

Neurons encode stimuli by emitting trains of actions potentials in response to sensory inputs. To uncover the corresponding neural code, the mapping between sensory inputs and output action potentials needs to be described with the help of a quantitative model [1]. In the recent years, generalized linear models have become a popular class of models for that purpose [2]–[4]. The most basic version of these models is the linear-nonlinear (LN) cascade, in which the instantaneous firing rate of the neuron is estimated by applying to the sensory signal successively a linear temporal filter and a static non-linear function. Phenomenological models of that kind are attractive because they are simple and efficient, and yet allow for enough freedom to fit experimental data. A drawback of this approach is however a lack of a direct relationship between the parameters of a LN cascade and the underlying biophysics, and it has been debated to what extent such descriptions capture the temporal dynamics of spike trains of real neurons [5], [6].

In more detailed models of the neural input-output mapping, membrane potential dynamics play the role of the intermediate between input currents and output action potentials [7]. While more biophysically faithful than linear-nonlinear models, these spiking neuron models are also significantly more complex and a significant amount of effort has been invested in reducing the dynamics of populations of spiking neurons to an effective mapping between their input and the output firing rate [8]–[17]. In the firing rate models (see [18] chapter 7.2 and [7] chapter 6), the input-output mapping of individual units is essentially a linear-nonlinear cascade, the linear filter being usually a simple exponential. Hence the two problems of relating a LN cascade to biophysical parameters and representing dynamics of spiking neurons by a firing rate model are very closely related.

In this communication, we examine to what extent a linear-nonlinear cascade can quantitatively reproduce the firing rate dynamics of spiking neuron models. To this end, we exploit known analytic results for integrate-and-fire models to obtain parameter-free expressions for the linear filter and static non-linearity. We then compare quantitatively the estimates of instantaneous firing rates obtained from various LN models with results from simulations of spiking neurons. For both the leaky integrate-and-fire (LIF) and exponential integrate-and-fire (EIF) models, in most of parameters space we find a good match between the estimate and the simulation results. In the case of the EIF, we show that a single exponential provides a good approximation for the linear filter, so that the LN cascade reduces to a firing rate model, the time constant of which we compute analytically. We then introduce an *adaptive timescale rate model* in which the decay time of the linear filter depends on the instantaneous firing rate, and show that this model provides a significant improvement with respect to both the basic rate model and the LN cascade. Finally, we examine a conductance-based spiking neuron and find that in this case also the adaptive timescale rate model provides an excellent description of firing rate dynamics.

## Results

**Figure 1:**
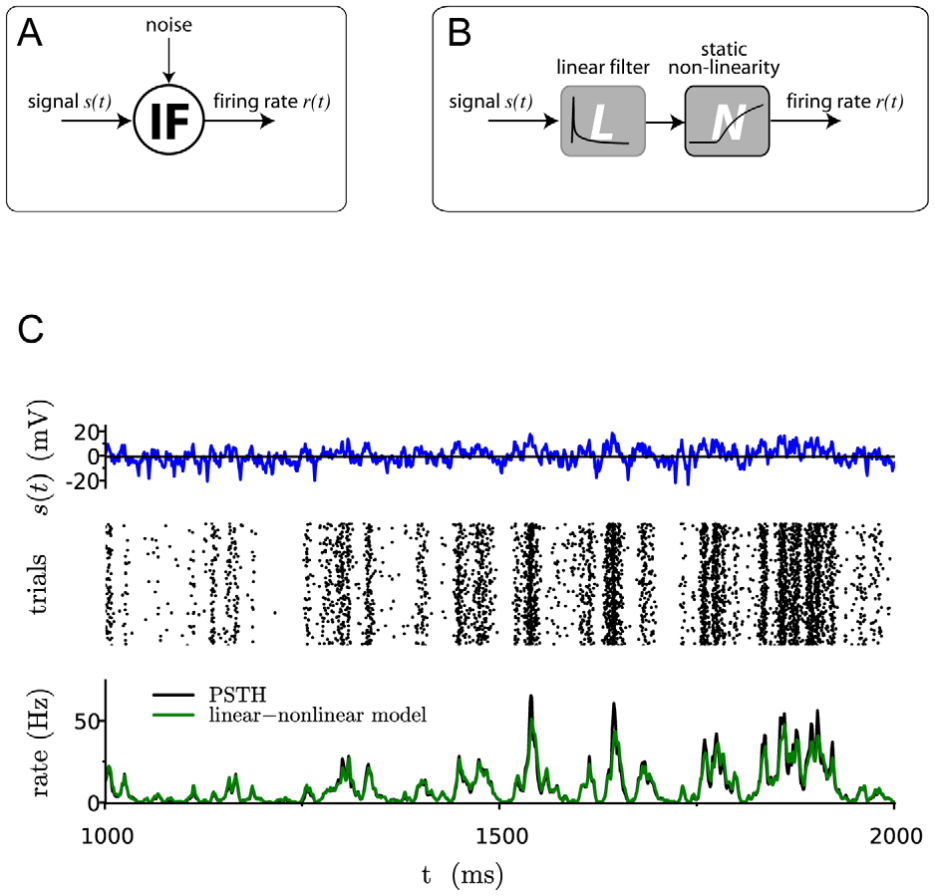
Comparing the input-output mapping of a spiking neuron to a linear-nonlinear cascade. A: A spiking neuron receives an input current consisting of a signal component that is identical in all trials and a noise component that is uncorrelated from trial to trial. Averaging trains of action potentials across trials gives a time-dependent output firing rate. B: Our aim is to obtain an estimate of the output firing rate by applying to the input signal a linear temporal filter followed by a static non-linearity. C: Illustration in the case of an exponential integrate-and-fire model. From top to bottom: input signal; raster plot of action potentials in a subset of 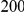 trials; comparison between the instantaneous firing rate and the output of the linear non-linear model.

We model a typical setup in which a given stimulus is repeatedly applied to a preparation, and action potentials of a neuron are recorded over many trials. We represent this neuron as a spiking neuron (either integrate-and-fire or conductance based) receiving a time-varying input. Here we consider only the case of input current, but our results could be easily extended to an input conductance. This current is assumed to consist of a sum of two components: an *input signal*, i.e. a time-varying input that is identical in all trials, and a *background noise* that is uncorrelated from trial to trial. The input signal can be interpreted as a feed-forward input from sensory pathways responding reliably to a stimulus which is identically repeated over trials, while the noise component represents the background activity of the surrounding network and inputs from other areas not directly controlled by the stimulus. Because of the noise, the spiking output varies from trial to trial. We therefore represent the output by its Peri-Stimulus Time Histogram (PSTH), i.e. the time dependent firing rate of the neuron [1], where the instantaneous firing rate is obtained by averaging over trials (see Fig. 1 and Materials and Methods). Equivalently, the PSTH can be interpreted as the firing rate of a large population of uncoupled neurons that all receive an identical input signal as well as background noise that is uncorrelated from neuron to neuron.

Our aim is to examine the extent to which the mapping between the input signal and the output firing rate can be approximated by a linear-nonlinear (LN) cascade consisting of two steps: (i) a linear temporal filter applied to the input signal; (ii) a static non-linear function applied to obtain the instantaneous firing rate (see Fig. 1). A standard method for determining the elements of a LN cascade is to use reverse correlations [19], [20], i.e. apply a signal with white-noise temporal statistics, compute the spike triggered average of the signal, which corresponds to the linear filter, and then determine the associated static non-linear function. Here we use a different approach: we exploit the known analytic results for integrate-and-fire neurons to infer the linear filter and the static non-linearity for particular limits of parameter values. Extending these expressions to the whole parameter space, we obtain the linear filter and static non-linearity in a parameter-free form. We then systematically assess the accuracy of the corresponding LN cascade by comparing its predictions for the firing rate with numerical simulations of spiking neurons.

To limit the available parameter space, we assume that the temporal statistics of the noise input are Gaussian with mean 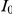, standard deviation 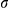 and no temporal correlation (white noise), while the temporal statistics of the signal input are Gaussian with zero mean, standard deviation 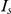 (which we refer to as the *amplitude* of the signal), and correlation time 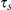 (colored noise). Because of background noise, in absence of signal (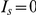), the neuron is not silent, but fires at a baseline rate 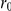 determined by 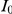 and 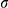. In this study, instead of varying 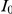, we vary 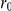, which is equivalent since all model neurons considered in this paper have continuous and monotonically increasing 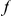-I curves.. The parameter space that we explore is thus four-dimensional and consists of 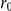, 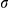, 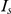 and 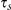.

### Determining the elements of the LN cascade

We wish to approximate the trial-averaged firing rate 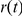 of the neuron using a linear-nonlinear cascade:
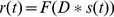
(1)where 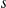 is the signal input, 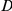 is a temporal linear filter, 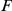 is a static non-linearity, and 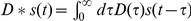 is the convolution between 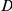 and 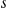. Moreover, 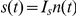, where 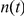 is a Gaussian process of zero mean, unit variance and correlation time 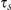.

The LN approximation of firing rate dynamics becomes exact in two extreme cases: (i) the linear limit of vanishing signal amplitude 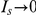; (ii) the adiabatic limit of very long signal correlation time, 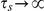. We first determine the linear filter 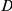 and the static non-linearity 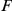 in these two limits. To extend the obtained expressions to the full parameter space, we simply set the linear filter 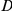 and the static non-linearity 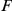 to be independent of the input parameters 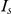 and 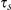, in which case the linear and adiabatic limits fully determine 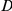 and 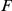. In this section we describe the general approach, which we later apply to specific neural models.

#### Linear filter

For a signal of vanishing amplitude 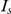, the variation of the firing rate 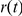 around the baseline firing rate 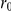 is small. Expanding Eq. (1) to linear order, the LN model reduces to
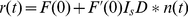
(2)where 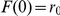.

On the other hand, in the linear limit the firing rate of the spiking neuron is given by
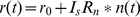
(3)where 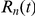 is the so-called rate response function of the neuron in presence of white noise [12], [21]–[23]. The Fourier transform of 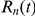 can be computed analytically for the leaky integrate-and-fire model [12], [24], [25], and for the exponential integrate-and-fire model an efficient numerical method has been designed to determine 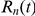 from the Fokker-Planck equation [26]. For completeness, the analytic derivation for the LIF is included in the Appendix.

Comparing Eqs. (2) and (3), it is straightforward to identify 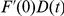 and 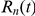. Note that 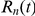 depends on the properties of background noise through 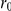 and 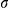.

#### Static non-linearity

In the limit 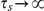, the input signal varies on a timescale that is much longer than the timescale of the linear filter, so that 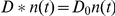, where 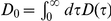, and in the LN approximation
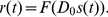
(4)


In the same limit, as the input signal varies slowly, at each point in time the neuron effectively receives a white noise input of mean 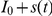 and standard deviation 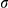. The response to such an input is given by the so-called 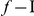 or transfer function 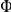 :
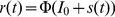
(5)


The transfer function for the LIF and EIF models receiving white noise is known analytically [27]. For the EIF an efficient numerical method has been designed to determine 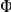 from the Fokker-Planck equation [26]. In both cases, the shape of 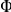 depends on the standard deviation 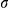 of background noise.

Comparing Eqs. (4) and (5) leads to the following identification:
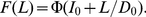
(6)


#### Extension to the full parameter space

For finite signal amplitude 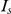 and correlation time 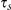, the LN cascade does not provide an exact description of firing rate dynamics, but only an approximation. Here we choose the linear filter 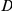 and the static non-linearity 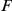 to be independent of the parameters of the signal 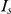 and 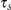. In that case, the two limits 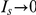 and 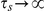 determine 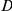 and 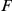 uniquely, up to multiplicative constants 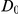 and 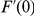. From Eq. (6), these two constants are constrained by 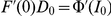. As the two constants enter the LN model only as a product, without loss of generality we set 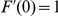, so that 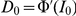. We therefore get:
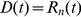
(7)

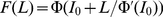
(8)where 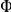 is the transfer function and 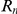 is the rate response function of the neuron. The units of 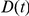 are Hz/mV, so that 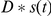 is the linear estimate of the firing rate in Hertz.

### Leaky integrate-and-fire neurons

We start by examining the leaky integrate-and-fire (LIF) model [28], in which action potentials are generated when the membrane potential crosses a fixed threshold value, the dynamics of the membrane potential being governed only by a leak current. Despite its apparent simplicity, this model is capable of reproducing quantitatively the transfer function of neocortical pyramidal cells in presence of *in vivo* like noisy inputs [29]. The LIF model has been studied extensively [30]–[32], and a number of analytic results are available for it. We first describe the linear filter and static non-linearity for the LIF model, and then assess the accuracy of the LN approximation for the input-output mapping.

#### Linear filter

As we set the linear filter 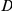 of our LN cascade to be independent of input parameters, 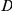 is given by the rate-response function of the model neuron. For the LIF model, the rate response function to oscillatory inputs in presence of white noise has been studied in [12], [24]. Its analytical expression is known in frequency (see Materials and Methods Eq. (31)), and we use the Fast Fourier Transform to obtain the values in time, as in [33]. The derivation of the rate response function is included in the Appendix. Some analytic results for 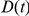 have been obtained for the limit 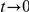 [34], [35]. In particular, in that limit, 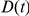 diverges as 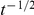, so that the LIF model is capable of responding very quickly to changes in the input signal.

**Figure 2:**
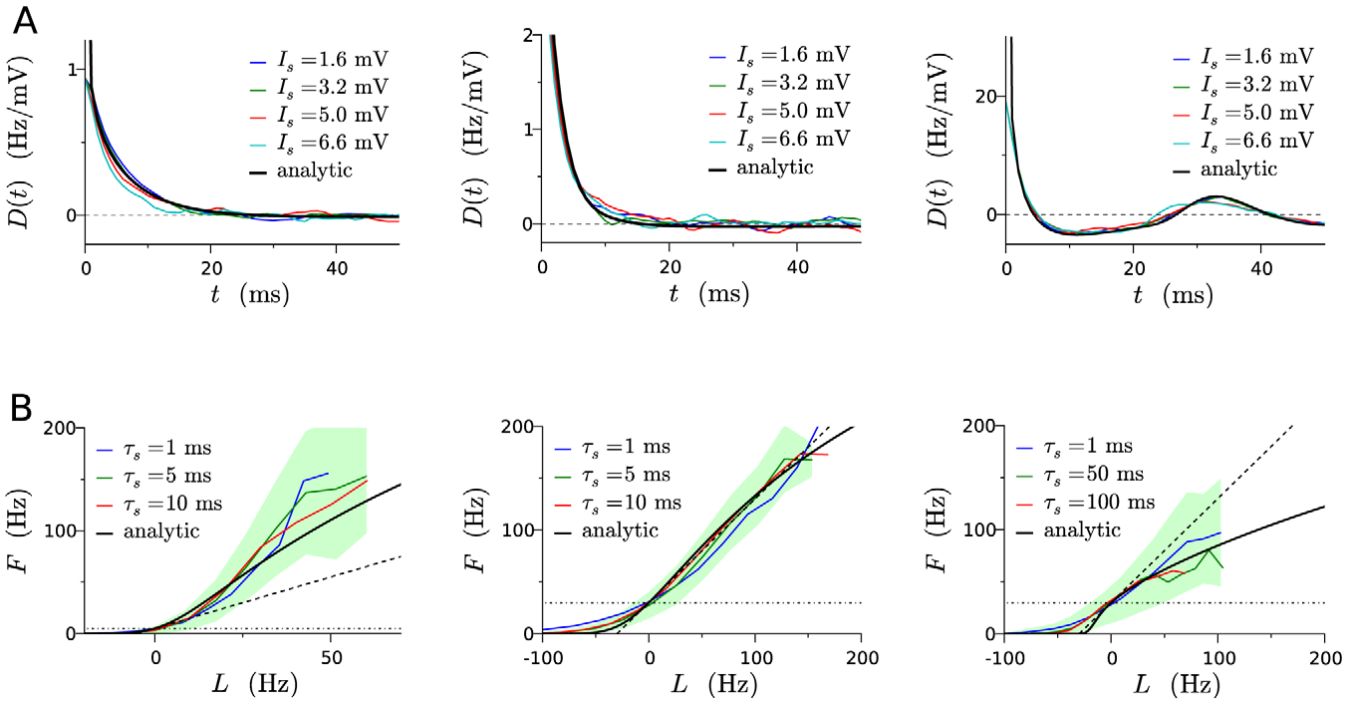
Linear filter and static non-linearity for the leaky integrate-and fire neuron, for three different sets of parameters for background noise. A: Analytic filter compared with the numerical spike triggered averages of the input signal, for three different amplitudes 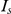 of the signal. The correlation time 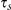 of the signal was set to 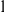 ms. B: Analytic non-linearity, compared with numerically estimated non-linearities for three different values of the correlation time 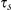 of the signal. The green area corresponds to deviations of one standard deviation around the mean for 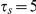 ms (green curve). The horizontal dotted line indicates the value of the baseline firing rate 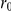 while the dashed line represents the tangent of unit slope. Going from left to right in the columns the parameters characterizing the background noise are: (i) 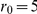 Hz, 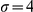 mV; (ii) 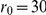 Hz, 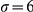 mV (iii)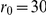 Hz, 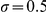 mV.

Background noise strongly modulates the response of the neuron [36], [37], so that both the timecourse and amplitude of the linear filter 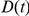 for 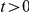 depend on the mean 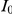 and standard deviation 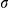 of the background noise, as shown in Fig. 2.

For strong background noise, 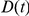 is a monotonically decreasing function, the decay time being of the order of a couple of milliseconds. This decay time depends on the mean 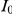 of the background noise, or equivalently the baseline firing rate 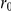 of the neuron [33]. For 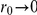, 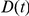 becomes proportional to the sub-threshold filter 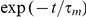 (this result was not reported in previous works, see Materials and Methods for the derivation), the decay time of 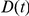 is thus given by the membrane time constant (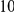 ms in our case). As 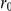 is increased, the decay time decreases, so that it is always faster than 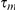.

For weak background noise, 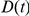 displays oscillations, at a frequency approximately given by the baseline firing rate 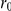 (see Fig. 2). The decay time of 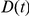 in this case is much longer (of the order of tens to hundreds of milliseconds), and it increases as the standard deviation 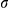 of background noise is decreased.

The amplitude of the linear filter approximately scales as the inverse of the standard deviation 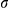 of background noise, so that the amplitude of the firing rate modulation in response to an input signal of a given amplitude 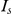 strongly depends on the amplitude of background noise.

It is interesting to compare the analytic linear filter 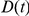 with linear filters obtained numerically using reverse correlations analysis. In the simulations, we inject an input signal 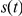 with very short correlation time (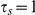 ms), and various amplitudes 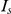, as well as a background noise of mean 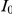 and standard deviation 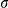. The numerical linear filter is then obtained by computing the spike-triggered average of 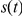 only. As shown in Fig. 2, our analytic filter matches well the numerical STAs, in absence of any fitting parameter. While this was expected for small 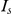, the match is good for any value of 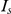. This fact, observed previously in [38], supports our choice of a linear filter independent of 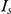.

#### Non-linearity

The analytic derivation of the transfer function 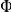 of the LIF in presence of white noise can be found in [27], and the expression is reproduced in Materials and Methods Eq. (27). For weak noise, 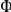 exhibits a sharp threshold, above which it is concave. As the standard deviation of noise is increased, the threshold is replaced by an inflexion point.

The static non-linearity 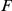 is obtained by rescaling the transfer function 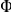 of the neuron, in such a way that the firing rate at the origin is equal to the baseline firing rate 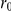, and the slope is unity (see Eq. (8)). As shown in Fig. 2, depending on whether 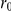 lies in the convex or concave region of 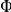, the non-linear function 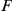 is either super-linear or sub-linear. Depending on the parameters of background noise, 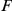 can thus display qualitatively different behaviors.

To compare our static non-linear function 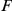 with the static non-linearity that would be obtained using a reverse correlation analysis, we follow the standard method [18]: we first compute the linear estimate 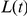 of the instantaneous firing rate (in bins of 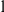 ms); for each bin, we then compare the linear estimate with the actual firing rate obtained from simulations. For a given value of 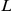, there is a distribution of actual firing rates. The numerical non-linearity is given by the average of this distribution as function of 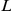. The width of the distribution, which can be significant, is not due to statistical noise in the data, but rather to the limitations of the LN model, as this distribution corresponds to variations in the output firing rate that cannot be accounted for by a linear-nonlinear transformation of the signal. In Fig. 2, the numerical non-linearities are compared with the non-linear function 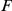 obtained from the transfer function, for different values of the signal correlation time 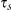. While a good match is expected for very large 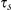, 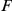 is close to the reverse-correlation estimates even for small 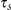. This observation supports our choice of 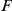 independent of the signal correlation time 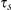.

#### Accuracy of the LN approximation for the rate dynamics

Once the linear filter and the static non-linearity are determined, we are in position to compare the estimate of the instantaneous firing rate provided by the LN cascade with the actual, numerically determined firing rates for different points in parameter space.

**Figure 3:**
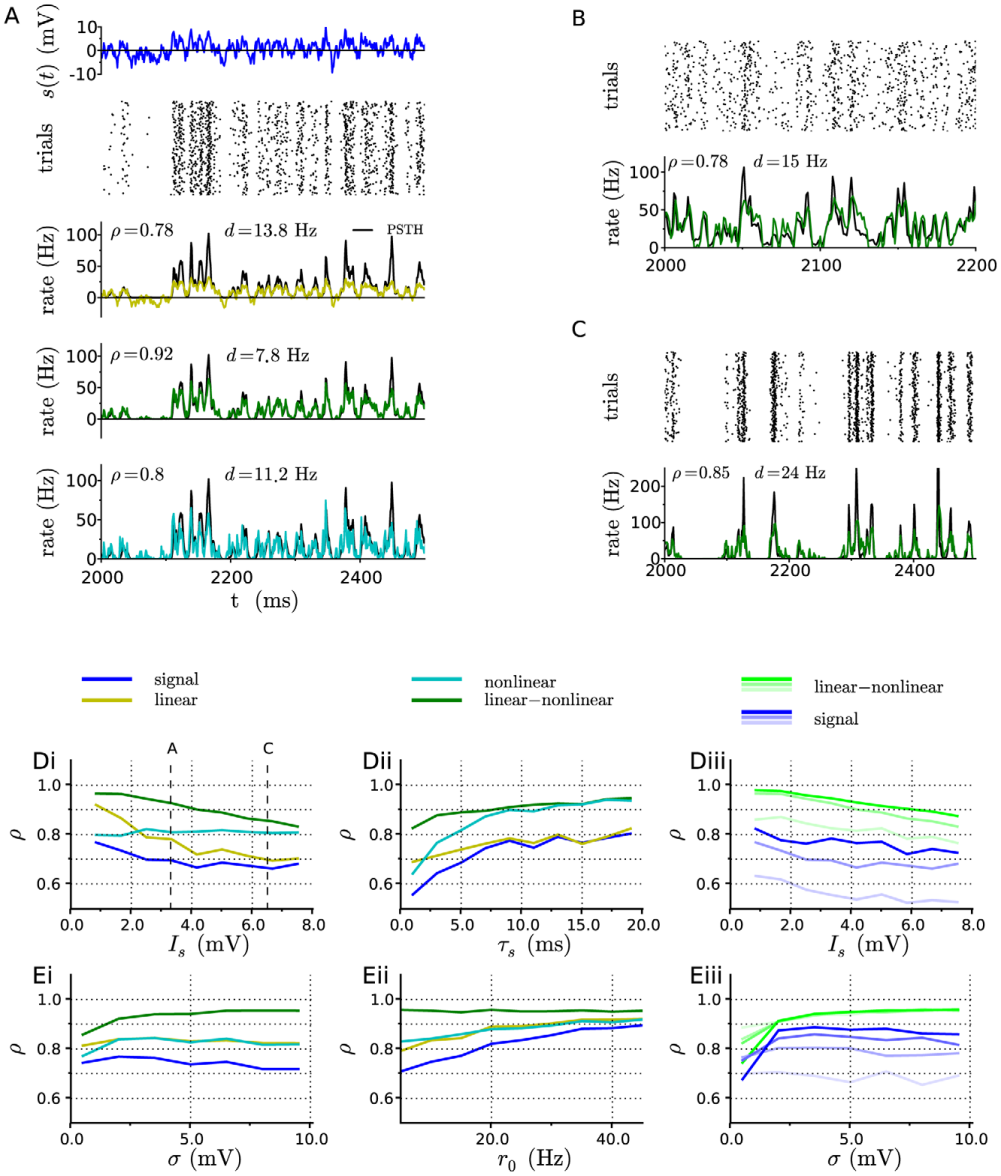
Comparison between estimates of the firing rate and numerical simulations, for the leaky integrate-and-fire model. A: Illustration for a given set of parameters (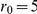 Hz, 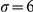 mV, 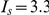 mV, 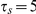 ms). From top to bottom: input signal trace; raster of action potentials in a subset of 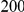 trials; comparison between the numerical PSTH (black) and the linear, LN, and nonlinear estimates. B: Comparison between PSTH and LN estimate at low noise (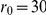 Hz, 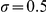 mV, 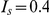 mV, 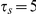 mV). C: Comparison between PSTH and LN estimate at very strong input signal (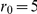 Hz, 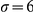 mV, 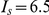 mV, 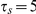 mV). In panels A–C, the values of 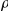 and 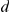 indicate respectively the correlation coefficiant and root-mean-square distance between the predicted firing rate and the numerical PSTH. D: Correlation coefficient between the numerical PSTH and various estimates as the amplitude 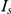 and correlation time 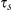 of the signal are varied, for fixed background noise parameters (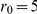 Hz and 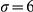 mV). From left to right: (i) 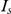 is varied for 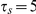 ms; (ii) 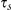 is varied for 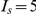 mV; (iii) 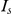 is varied for three increasing values of 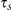 (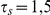 and 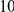 ms), shown with curves of increasing darkness. E: Correlation coefficient between the numerical PSTH and various estimates as the baseline firing rate 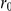 and the amplitude 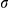 of the noise input are varied, for input signal parameters 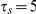 ms and 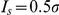. From left to right: (i) 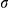 is varied for 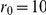 Hz; (ii) 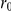 is varied for 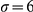 mV; (iii) data for four increasing values of 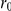 (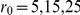 and 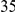 Hz) is shown with curves of increasing darkness.

Fig. 3 A illustrates the comparison between the numerical PSTH and three different approximations: (i) the linear approximation, in which only the linear filter is applied to the signal; (ii) the nonlinear approximation, in which only the static non-linearity 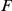 is applied to the signal; (iii) the full LN cascade. The main qualitative differences between the three approximations can be clearly seen in Fig. 3 A. In the linear approximation, the firing rates can take negative values; applying subsequently the non-linearity corrects for this, and further amplifies or attenuates the firing rates depending on whether the non-linearity is super-threshold or sub-threshold. In the purely non-linear approximation, the firing rate depends only on the instantaneous value of the signal, so that the firing rate fluctuates too fast compared to the PSTH. Applying a linear filter corrects for that. Note that in this example, 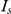 is large enough, and 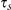 short enough, so that the instantaneous firing rate has very large fluctuations (between 0 and 100Hz) on fast time scales. Thus, we are far from the parameter ranges where one would expect the approximations made to derive filter and non-linearity to hold.

The degree to which various approximations match the numerical PSTH clearly depends on the parameters of the input signal and background noise. To get a quantitative comparison, we computed the Pearson's correlation coefficient 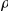 between the estimates and the numerical PSTH, as well as the root mean square (RMS) distance 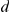 between both, for various values of the parameters (see Methods for pros and cons of the two measures). The correlation coefficient is 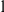 in case the two traces are identical, and 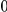 in case the traces are fully uncorrelated, the precise value depending only on the temporal dynamics of the traces and not on their mean and standard deviation. To interpret the measured values of 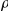, we also compute the correlation between the signal and the PSTH, which corresponds to the performance of an estimate of the firing rate obtained by simply rescaling the mean and variance of the signal. For example, in Fig. 3A, the correlation between signal and PSTH is 0.78 (corresponding to 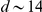
Hz) while the correlation between the LN model and the PSTH is 0.92 (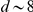
Hz). This means that in this example, values of 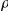 of order 0.8 correspond to poor approximations of the PSTH, while values of order 0.9 or more represent significant improvements compared to a trivial rescaling of the signal.

Fig. 3 displays the performance of different models as the amplitude 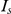 of the signal is increased, the other parameters being held constant. For 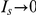, the linear and LN models become identical. In that limit, the correlation coefficient 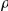 between the models and the PSTH approaches 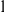, but does not strictly reach this limit because of statistical fluctuations in the data: for 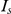 small, the signal-induced variations in the output firing rate are small, and become comparable to the fluctuations in the numerical PSTH that are due to the finite number of trials used for averaging.

As 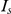 is increased, large values of the input signal lead to increasingly precise and reliable emission of action potentials. As a consequence, the variations of the output firing rate become larger, and the non-linearity plays an increasingly important role. The accuracy of the LN model progressively deteriorates, but 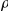 remains above 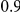 far into the non-linear regime. As an illustration, Figs. 3 A and C display the comparison between the LN estimates and the numerical PSTHS for two values of input signal amplitude 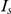, all other parameters being identical. The accuracy of the purely non-linear model is approximately independent of 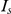, but relatively low (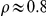). It is only in the limit of very strong signal amplitude that the accuracy of the non-linear model becomes comparable to the LN model.

The correlation between the signal and the output increases as the correlation time of the signal is increased (Fig. 3). In parallel, the accuracy of the LN model also increases, but to a relatively smaller degree.

Although the non-linear filter and static non-linearity depend on the parameters of background noise (see Fig. 2), the performance of the model varies weakly with the baseline firing rate 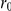 and noise amplitude 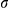. The main exception is the limit of small 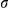 in which none of the models significantly improves over the correlation between the signal and the output (see Fig. 3 B for an illustration). The reason for this is that for weak noise 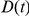 displays oscillations at long timescales: the input signal at a given time influences the output rate on a range of different timescales, and the non-linear interferences between these timescales are not well described by a linear-nonlinear cascade. However, the LN approximation performs well already for 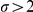 mV, the correlation coefficient between the LN estimate and the PSTH being larger than 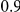. This correlation coefficient further increases with increasing 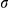, while it appears to be independent of the baseline firing rate 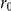.

In summary, the linear-nonlinear model of input-output mapping provides a good approximation of the firing rate dynamics for most of the parameter space, two notable exceptions being the limit of weak background noise (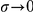) and the limit of very large input signal (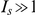).

### Exponential integrate-and-fire neurons

The exponential integrate-and-fire (EIF) model [21] is a generalized integrate-and-fire model in which the action potentials are generated by an exponential current instead of a fixed threshold. It is the simplest model capable of reproducing membrane potential dynamics and action potential times of cortical neurons in presence of a noisy input, and it has been used to successfully fit data from layer 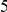 pyramidal cells [39], interneurons [40] as well as cerebellar Purkinje cells [41]. In this section, we examine an EIF neuron with parameters corresponding to pyramidal cells (see Materials and Methods).

#### LN cascade

**Figure 4:**
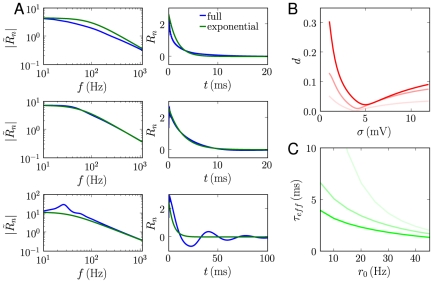
Linear filter and single timescale approximation for the exponential integrate-and-fire model. A: Comparison between the full filter and the single timescales approximation for three different values of noise amplitude 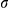 and fixed baseline firing rate 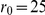 Hz. From top to bottom: 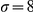 mV, 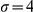 mV and 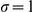 mV. Left column: linear filter in frequency; right column: linear filter in time. B: Root-mean-square distance 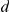 between the Fourier transform of the linear filter and the single timescale approximation, as function of noise amplitude 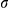. Data for three values of 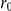 (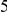, 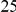, and 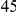 Hz) is shown with lines of increasing contrast. C: The effective timescale 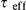 obtained from the single timescale approximation, as function of baseline firing rate 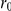. Data for three values of 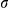 (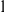, 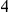, and 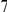 mV) is shown with lines of increasing contrast.

The linear filter 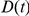 and the static non-linearity 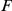 for the EIF model are qualitatively similar to the LIF model, a crucial difference being that for the EIF model 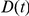 remains finite in the limit 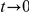, and therefore exhibits slower timescales. The influence of background noise on 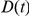 is qualitatively comparable to the LIF model: for large background noise 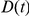 decays monotonically, while for weak background noise it displays oscillations (see Fig. 4). Depending on the parameters of background noise, the static non-linearity displays the super-linear and sub-linear regimes, as in Fig. 2.

**Figure 5:**
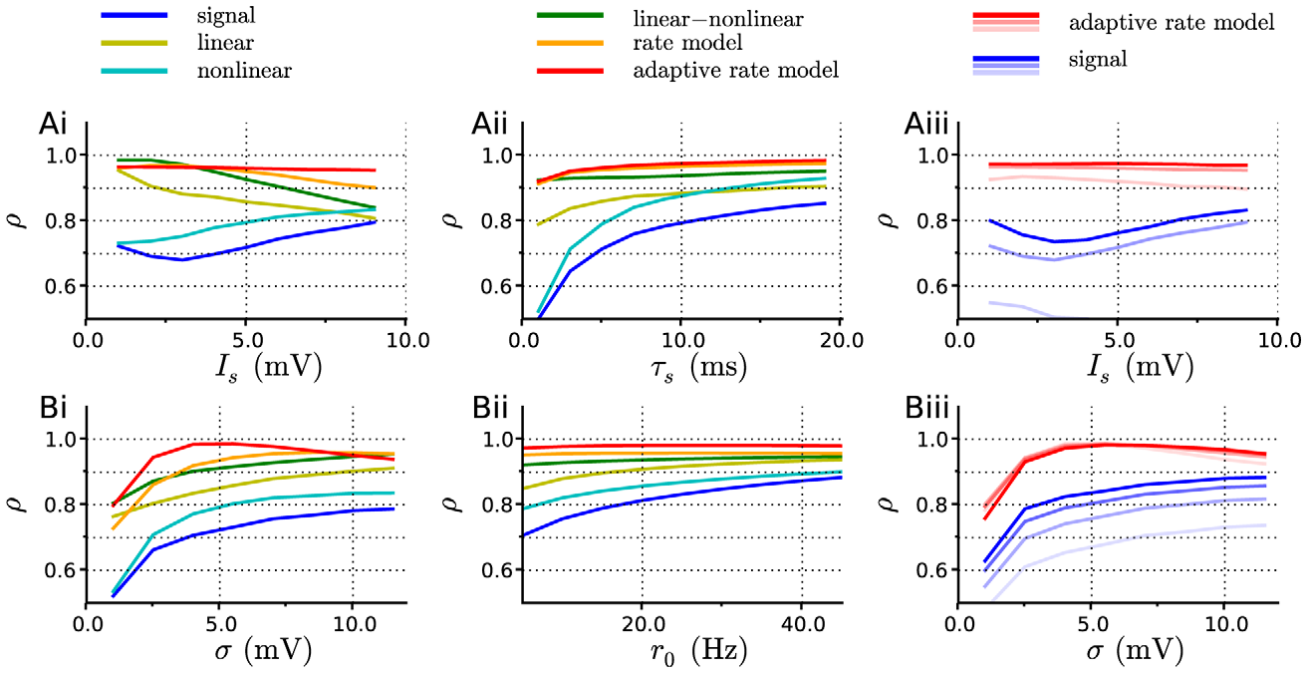
Correlation coefficient between the numerical PSTH and various estimates of the firing rate, for the exponential integrate-and-fire model. A: Effect of varying parameters 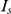 and 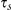 of the input signal, for 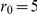 Hz and 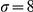 mV. From left to right: (i) the signal amplitude 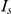 is varied for a correlation time 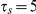 ms; (ii) 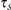 is varied for 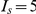 mV; (iii) 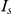 is varied for three increasing values of 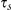 (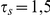 and 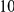 ms), shown with curves of increasing darkness. B: Effect of varying parameters of the background noise for 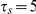 ms and 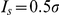. From left to right: (i) The amplitude of background noise 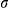 is varied for 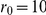 Hz; (ii) 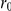 is varied for 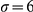 mV; (iii) data for four increasing values of 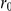 (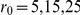 and 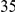 Hz) is shown with curves of increasing darkness.

Fig. 5 displays the performance of the linear, nonlinear and LN approximations as a function of the parameters of input signal and background noise. Similarly to the LIF model, the LN cascade provides a good approximation of the firing rate dynamics in most of the parameter space except for the limits of weak background noise and very large signal amplitude.

#### Rate model

In the case of the EIF model, as the linear filter 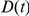 remains finite in the limit 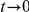, it is possible to exploit the known asymptotic results to derive a simpler analytic approximation for the firing rate dynamics, in which the linear filter is exponential with a single, effective timescale 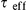:
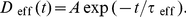
(9)


With such an exponential filter, the linear non-linear cascade of Eq. (1) can be rewritten as
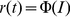
(10)

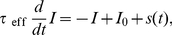
(11)so that the dynamics of the firing rate are given by a rate model (see [18] chapter 7.2 and [7] chapter 6).

To derive an analytic expression for the timescale 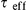 of the equivalent exponential filter, we note that the amplitude of the Fourier transform of 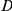 decays as 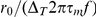 for large frequencies 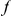, while for vanishing frequencies it reaches a finite value equal to the slope 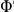 of the transfer function 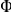, evaluated at the baseline firing rate 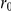 and background noise amplitude 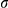 [21]. Matching these asymptotics to those of the Fourier transform 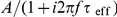 of an exponential filter, we get:
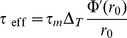
(12)

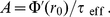
(13)


To compare quantitatively the full linear filter 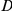 with the approximate exponential filter 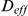, in Fig. 4 we display the root mean square distance 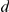 between the Fourier transforms of 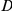 and 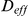, as a function of the baseline firing rates 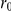 and noise amplitude 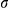. Independently of 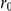, 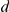 displays a minimum as function of 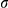, at a location 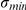 close to 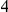 mV for small 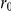. Around that minimum, the exponential filter provides an excellent approximation of the full filter. For very small 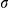, the exponential approximation clearly fails to reproduce the oscillations in 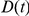.

As shown in Fig. 4 C, the value of 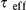 is a decreasing function of the baseline firing rate 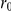, and a decreasing function of the noise amplitude 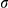 (Fig. 4). The effective timescale 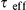 is faster than the membrane time constant 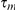 (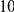 ms in our case) in most of parameter space. The only exception is the limit of small 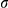, in which the exponential filter is a bad approximation of the full linear filter, so that values of 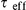 larger than 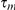 are an artifact of the approximation.

**Figure 6:**
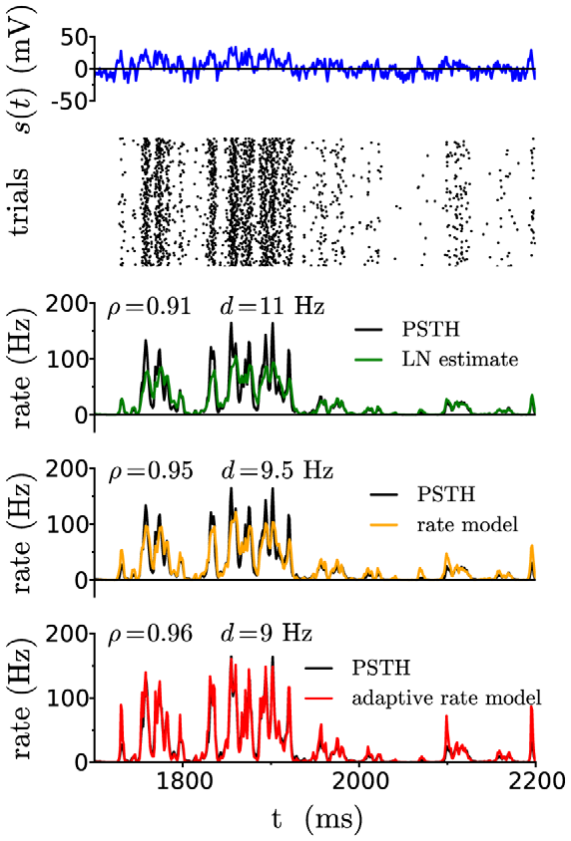
Comparison between estimates of the firing rate and numerical simulations, for the exponential integrate-and-fire model. Illustration for a given set of parameters (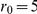 Hz, 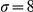 mV, 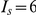 mv, 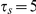 ms). From top to bottom: input signal trace; raster of action potentials in a subset of 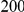 trials; comparison between the numerical PSTH (black) and the LN estimate, the rate model and the adaptive timescale rate model. The values of 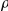 and 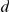 indicate respectively the correlation coefficiant and root-mean-square distance between the predicted firing rate and the numerical PSTH.

Fig. 6 illustrates the comparison between a numerical PSTH and the estimate obtained from the rate model. Fig. 5 displays the accuracy of the rate model approximation, quantified by the correlation coefficient between the estimate and the PSTH, as a function of the parameters of the input signal and background noise. In most of the parameter space, the accuracy of the rate model is comparable to the performance of the LN approximation in which the full filter is used. For some values of the parameters, the rate model appears to outperform the full LN cascade. This happens when the exponential filter is faster than the full linear filter, in which case the rate model predicts large firing rate transients better than the full LN model, but overestimates small transients in contrast to the full LN cascade. When the input signal amplitude is large, large transients dominate in the value of 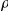, so that the rate model leads to larger values of 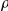 than the full cascade.

Similarly to the full LN model, the performance of the rate model degrades in the two limits of weak background noise and very large input signal amplitude. The advantage of the rate model over the full LN cascade is its simplicity, which allows for a very efficient and robust implementation.

#### Adaptive timescale rate model

While the LN and rate models provide good estimates of firing rate dynamics in most of parameter space, their accuracy deteriorates as the amplitude 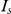 of input signal increases. To improve the firing rate estimates in that parameter regime, we introduce *adaptive* models, in which the linear filter is allowed to change over time.

So far, the linear filter 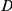 and static non-linearity 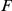 used in various approximations were evaluated at the baseline firing rate 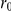 and held constant in time. However, for large input signal amplitude, the instantaneous firing rates deviate strongly from 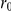. As noted previously, the timescale of 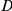 decreases with increasing 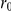, hence at high 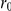 the neurons respond faster to inputs, but the LN model does not incorporate this effect. To improve the estimates of firing rate at large 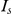 it seems therefore natural to replace at every timestep the static linear filter 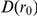 by the linear filter corresponding to the instantaneous firing rate 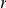 (i.e. the estimate of the firing rate at the previous time step). We call such a model an adaptive linear-nonlinear model as at every timestep the linear filter is matched to the instantaneous firing rate. With such a modification, the firing rate response becomes faster for larger input currents.

In the adaptive LN model, the linear filter has to be computed in principle at every timestep by integrating the Fokker-Planck equation, which is computationally cumbersome. Instead, for the EIF model, at every timestep we approximate the instantaneous filter by the corresponding exponential filter (see Eq. 9). We thus obtain an *adaptive timescale rate model*:
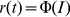
(14)

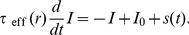
(15)


In this model the timescale 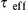 depends implicitly on time through the instantaneous firing rate, and can be obtained from
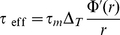
(16)where 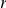 is the instantaneous firing rate (i.e. the estimate of the firing rate at the previous time step).

Fig. 6 illustrates the comparison between a numerical PSTH and the estimate obtained from the rate model. When the instantaneous firing rates are high, the adaptive-timescale rate model provides a better estimate than the non-adaptive models. For small firing rates, the adaptive-timescale model overestimates some transients; this effect is due to the fact the the effective timescale 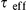 at low rates is faster than the timescale of the full linear filter 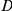.

As shown in Fig. 5 B, in contrast to non-adaptive models, the accuracy of the adaptive-timescale rate model does not degrade with increasing input signal amplitude, but remains constant, the correlation coefficient 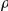 between the numerical PSTH and the estimate being greater than 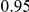. Overall, the adaptive-timescale rate model thus provides an excellent estimate of the firing rate dynamics in most of parameter space. In particular, as function of background noise amplitude 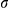, 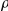 displays a broad maximum where it reaches values of 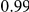 (cf. Fig. 6). The accuracy of the adaptive-timescale rate model deteriorates in the limit of very small noise amplitude.

### Conductance based model

So far we examined only models of the integrate-and-fire type, which are one-dimensional in the sense that action potential generation is controlled by a single variable, the membrane potential. In contrast, in biophysically more detailed models, the dynamics of the membrane potential are coupled to the dynamics of a number of ionic conductances, so that these models have higher dimensionality. In spite of this additional complexity, we will show that our results can be easily extended to a standard conductance-based model of Hodgkin-Huxley type, the Wang-Buzsáki model [42].

Studying the dynamics of conductance-based models in the presence of noise is in general very challenging, and the transfer and linear response functions are in general not known analytically. It has however been found that the exponential integrate-and-fire model with appropriately chosen threshold, reset, spike sharpness and refractory period closely reproduces the transfer and linear response functions of the Wang-Buzsáki model [21]. Although the values of these four parameters were chosen so as to reproduce the scaling of the transfer function around threshold and at strong inputs, the linear response functions of the Wang-Buzsáki and EIF models also match for any value of input parameters. In the original study this was observed with the help of direct numerical simulations of both models. We evaluated the transfer function and linear response function of the EIF model using the direct integration of Fokker-Planck equation [26] and comparing with simulations of the Wang-Buzsáki models we confirm the previously observed close match.

**Figure 7:**
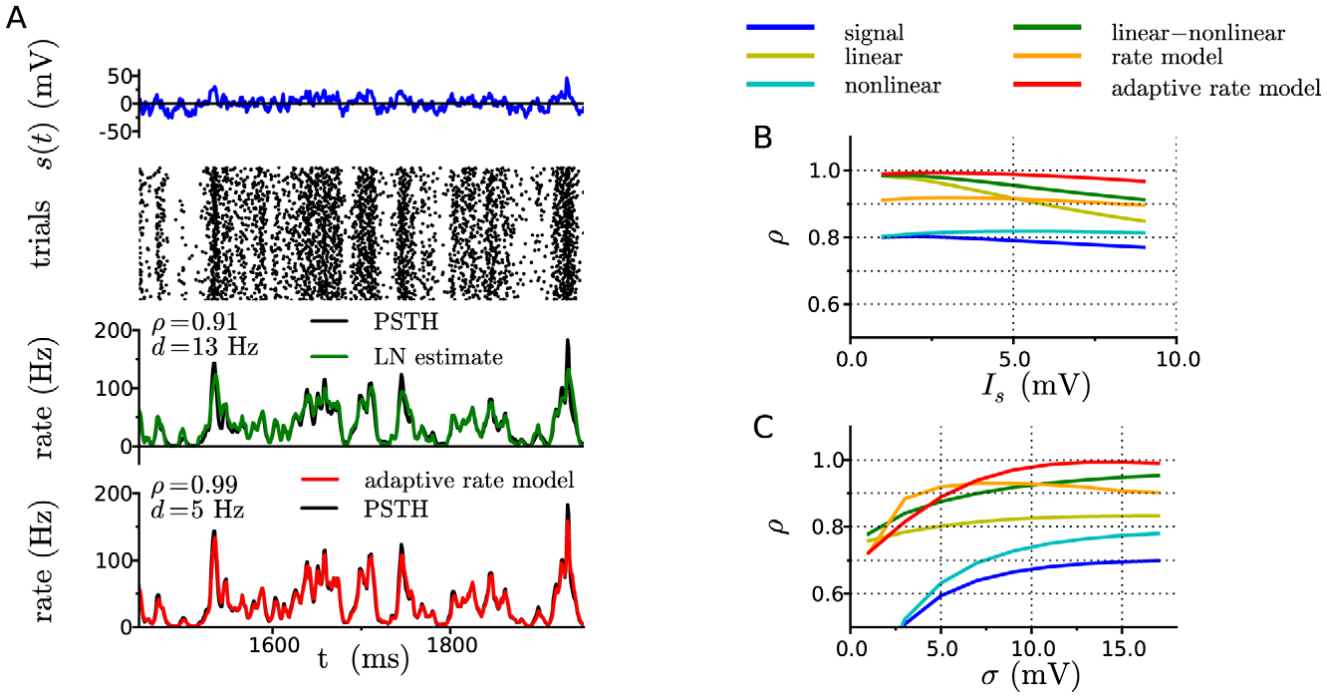
Comparison between estimates of the firing rate and numerical simulations, for the Wang-Buzsáki model. A: Illustration for a given set of parameters (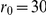 Hz, 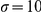 mV, 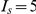 mV, 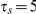 ms). From top to bottom: input signal trace; raster of action potentials in a subset of 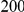 trials; comparison between the numerical PSTH (black) and the LN estimate and the adaptive-timescale rate model. The values of 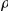 and 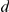 indicate respectively the correlation coefficiant and root-mean-square distance between the predicted firing rate and the numerical PSTH. B: Correlation coefficient between the numerical PSTH and various estimates of the firing rate as the amplitude 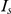 of the input signal is varied. The values of other parameters are 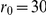 Hz, 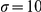 mV, and 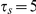 ms. C: Correlation coefficient between the numerical PSTH and various estimates of the firing rate as the amplitude 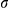 of background noise is varied. The values of other parameters are 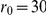 Hz, 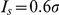 and 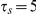 ms.

The linear filter and static non-linearity for the Wang-Buzsáki model can thus be directly obtained from the transfer function and linear response function of the EIF model with appropriate parameters (see Materials and Methods). Fig. 7 illustrates the comparison between the firing rates obtained from the LN approximation, and numerical simulations of the full conductance-based model. As for the EIF model, the match between the LN estimate and the numerical PSTH is good. Moreover, the simplified rate models developed for the EIF model carry over to the Wang-Buzsáki model, and provide simple approximations for the rate dynamics using the transfer function alone. In particular, the adaptive-timescale rate model leads to very high correlation coefficients between the estimated firing rate and the numerical PSTH (cf. Fig. 7). Note that such a high value of 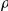 can be somewhat misleading: in Fig. 7 A the correlation coefficient of 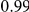 corresponds to a root-mean square distance of 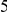 Hz between the predicted firing rate and numerical PSTH. This is significantly larger than the error in the PSTH, which is for these parameters of the order of 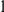 Hz (see Materials and Methods).

## Discussion

In this study, we examined the ability of phenomenological models to describe the firing rate output of spiking neurons in response to a time-varying input signal that the neurons receive on top of background synaptic noise. The phenomenological models we considered belong to the class of linear-nonlinear cascade models: the firing rate is estimated by first applying a linear filter to the input signal and then correcting for deviations from linearity using a static non-linear function. Instead of using a fitting procedure, the linear filter and static non-linearity were obtained in a parameter-free form by exploiting analytic results valid for particular limits of input signal parameters. This approach allowed us to systematically quantify the accuracy of the phenomenological models by comparing their predictions with results of numerical simulations of spiking neurons.

We found that linear non-linear models provide a quantitatively accurate description of firing rate dynamics of leaky integrate-and-fire, exponential integrate-and-fire and conductance-based models, as long as the background noise is not excessively weak. In the limit of vanishing variance of background noise, the spiking of neurons exhibits locking to the input signal [43] that cannot be accounted for by the linear-nonlinear cascade. The accuracy of the cascade models also decreases as the amplitude of the input signal is increased, but this effect becomes quantitatively important only for very strong input signals that lead to instantaneous firing rates in the range of hundreds of hertz. As methods for computing analytically the linear filter and static non-linearity are available only for white noise background inputs, we have not systematically studied the situation in which the background noise is colored. However, one could in principle compute numerically both linear filter and static non-linearity, and then use the same approach as introduced here.

For the exponential integrate-and-fire and conductance-based models, the linear filter can be accurately approximated by a single exponential in a large range of noise amplitudes, so that the linear-nonlinear model can be reduced to a firing rate model. We obtained a simple analytic expression for the time constant of the rate model, directly relating it to the biophysical parameters of the neuron. The value of the time constant in particular depends on the sharpness of action potential initiation and the baseline firing rate of the neuron.

Interestingly, the EIF model is essentially the only non-linear integrate-and-fire model that can be described by such a simple rate model, since it is the only model in this class whose firing rate response decays as 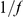 in the high frequency limit, independently of whether the background noise is white or colored [21]. The firing rate response of LIF neurons decay as 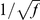 in the case of white noise [12], [24], which makes it impossible to reduce the LIF model to a simple rate model. In the case of colored noise, the response stays finite in the high frequency limit [12]. This fact makes it possible to approximate the LIF model with colored noise by an even simpler rate model, in which the firing rate depends instantaneously on the input through the 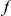-I curve (the ‘nonlinear’ model described here, see also [15], [44]). A comparison of the predictions of the nonlinear model with simulations of the LIF neuron with colored noise however showed that the accuracy of the nonlinear model did not improve significantly with respect to the white noise case (data not shown), presumably because the response function of LIF model does not become totally flat as the noise correlation time is increased [44]. Finally, the firing rate response of QIF neurons decays as 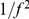. This model could therefore be approximated by a two-variable rate model, in order to reproduce this high frequency behavior.

Finally, we introduced a simple generalization of the rate model in which the time constant depends on the instantaneous firing rate of the neuron. This *adaptive-timescale rate model* reproduces with a high precision the firing rate dynamics of exponential integrate-and-fire and conductance-based models, even for input signals of very large amplitude. This model can be extended to the case of colored noise, and its accuracy degrades gracefully as the correlation time of the background noise is increased (data not shown).

### Comparison with previous studies

Phenomenological firing-rate models (and the closely related neural field models) are basic tools of theoretical neuroscience, and several earlier studies have looked for quantitative mappings between such models and more biophysically detailed, spiking neuron models. To our knowledge, our study is the first to compare extensively across parameter values the output of a phenomenological rate model to the firing rate dynamics of spiking neurons.

The question of how to reduce the firing rate dynamics of populations of spiking neurons to simplified ‘firing rate’ models has been the subject of numerous previous studies. Most reductions however ignore the single cell dynamics and eventually end up with rate equations in which the only time scale is a synaptic time scale (see e.g. [45]). Such a reduction can be performed rigorously in the limit in which the dynamics of the synapses are slow [9], [46]. Another approach is to use another slow variable, e.g. an adaptation variable, as the only dynamical variable (see e.g. [15]). The drawback of this type of approach is that one can only capture the dynamics on the slow time scales, and all the fast time scales related to spiking dynamics are lost. Shriki et al (2003) reduced the dynamics of a network of specific conductance-based model neurons to firing rate dynamics, but their approach is based on numerical fits of both the static non-linearity and of the dynamical firing rate response.

The correspondence between linear-nonlinear cascade models and spiking neuron models has been examined in several earlier works. In [47], [48], techniques were developed for computing the linear filter and static non-linearity for integrate-and-fire models, while similar questions for the Hodgkin-Huxley model were addressed in [49], [50]. In these works, the authors consider the situation in which background noise is absent, so that the neuron does not fire spontaneously in absence of input signal. In our framework, this corresponds to the double limit of 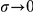 and 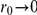. The limit of periodically firing neurons, i.e. vanishing noise but non-zero firing rate, was investigated in [51]. In [16], linear-nonlinear cascade models were used to approximate the firing rate dynamics of a spike response model with escape noise [7]. In contrast, here we examined integrate-and-fire and conductance-based models in presence of more biophysically realistic diffusion noise. Similarly to our case, in [7] the linear filter was determined analytically, however the static nonlinearity was obtained by fits to the data.

### Predicting full spike trains

To produce trains of action potentials, the linear-nonlinear cascade model is often supplemented by a third step, a stochastic Poisson process which at every time step generates an action potential with a probability given by the instantaneous firing rate obtained from the cascade. In this study, we have not attempted to compare the full statistics of spike trains produced by such a linear-nonlinear-Poisson model with the statistics of spike trains of integrate-and-fire neurons. Instead we have concentrated on the instantaneous firing rate, which is equivalent to the first-order statistics of spike trains. The instantaneous firing rate provides information about the timing of individual spikes, but does not specify the correlations between successive spikes in a given train. It has been argued that the refractory period and other post-spike effects play an important role in determining precise spike timing [5], [52]–[54].

To reproduce faithfully the full statistics of spike trains of spiking neurons, the linear-nonlinear cascade would have to be supplemented with post-spike history filters leading to correct higher order statistics. Several modeling approaches have been developed to include post-spike filters [54], [55], most prominently generalized linear models (GLMs) [3], [56] and spike-response models (SRMs) [7]. The main difference between these two classes of models is that in SRMs, the quantity obtained after applying the linear filters to the inputs and previous spikes is interpreted as the membrane potential, while no such assumption is made in GLMs. In consequence SRMs are usually fitted to intra-cellular recordings [57], while GLMs are more often applied to extra-cellular recordings [4], [56]. In both classes of models, because of post-spike filters, the firing rate is an implicit function of the input signal, while in conventional LN models as used here the firing rate is an explicit function of the input signal, a very desirable property (for details see [16], [58]). It is not clear how to generalize an LN cascade to take into account correlations in the spike train while preserving this property. It should be noted that the linear filter we use incorporates effects of refractoriness - this is most noticeable at low noise, where the filter exhibits oscillations due to effective refractoriness (see also [55]). While additional effects would need to be incorporated in post-spike filters, these filters should affect only the higher order statistics of spike trains, and not the instantaneous firing rates.

### Relationship with experimental data

A large number of studies have exploited linear-nonlinear models to fit experimentally measured data. In the majority of these studies [2], [4]–[6], [54], the linear-nonlinear model represents the mapping between the stimulus and neuronal firing, and therefore typically encapsulates several processing stages that transform the stimulus into a direct input to the neuron. In contrast, here we considered the mapping between the direct input to the neuron and its output. Such direct mappings have been studied experimentally *in vitro* [55], [59]–[63]. In these studies, the input-output mapping of cortical neuron was investigated in absence of background noise (note in particular that the spike-triggered average inputs display oscillations as in our low noise case). *In vivo* recordings indicate that background synaptic noise is a fundamental component of cortical processing [64], [65], as ongoing neural activity in higher cortical areas implies that only a part of the total input to a neuron can controlled by a sensory stimulus. More recent *in vitro* studies [22], [23], [29], [61] have therefore injected artificial background activity on top of the repeating signal. These studies have however mostly explored the linear regime, and it seems important to further examine the non-linear regime, varying systematically signal and noise parameters.

Exponential integrate-and-fire models have been used to predict individual action-potentials of cortical neurons, however post-spike adaptation currents had to be taken into account [39], [66], [67]. We therefore expect that the linear-nonlinear and rate models we developed here for the eIF model will have to be supplemented with additional adaptation components to reproduce accurately the firing rate dynamics of cortical neurons.

## Materials and Methods

### Integrate-and-fire models

In integrate-and-fire models, action potentials are generated solely from the underlying dynamics of the membrane potential [7]. These dynamics are given by [21]:
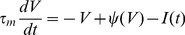
(17)where the membrane potential 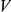 is determined with respect to the resting potential of the cell, 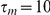 ms is the membrane time constant, 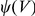 is a spike generating current, and 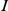 is the total current (expressed in mV) elicited by synaptic inputs to the neuron.

We studied two different versions of the integrate-and-fire model:

- *Leaky integrate-and-fire (LIF) model —* in this model, 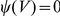, there is no spike-generation current, and an action potential (AP) is emitted when the membrane potential crosses a fixed threshold value 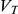. The membrane potential is subsequently reset to a value 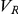 after a refractory period 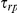. The values used in the simulations were 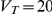 mV, 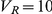 mV and 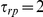 ms.
- *Exponential integrate-and-fire (EIF) model —* in this model, the spike generation current is exponential:
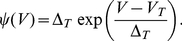
(18)

Once the membrane potential crosses the threshold 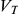, it diverges to infinity in finite time. This divergence represents the firing of an action potential. Following the divergence, the membrane potential is reset to a value 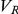 after a refractory time 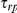. The parameter 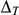 quantifies the sharpness of the AP initiation. The parameter values used in most of this study were 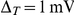, (a typical value for pyramidal cells [39]), 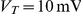, 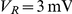 and 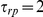 ms.

### Conductance based model

We used the Wang-Buzsáki model [42], which is a modified Hodgkin-Huxley model. The dynamics of the membrane potential are given by
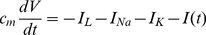
(19)where 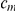 is the membrane capacitance (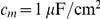), 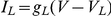 is the leak current (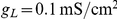;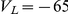 mV), 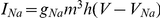 is the sodium current, 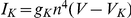 is the delayed rectifier potassium current, and 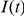 is the total synaptic input current.

The activation of the sodium current is assumed instantaneous:
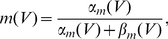
(20)while the kinetics of the gating variables 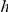 and 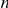 are given by:
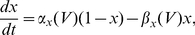
(21)with 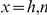.

The functions 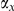 and 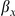 are given by:
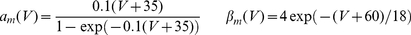
(22)

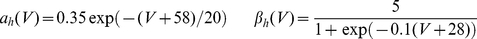
(23)

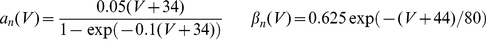
(24)


The maximum conductance densities and reversal potentials are: 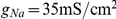, 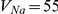 mV; 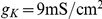, 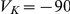 mV.

### Inputs to the neurons

As explained in the main text, in this study we assume that the synaptic inputs to the neuron are separated into two groups: (i) inputs that are identical across trials, and which we call the “signal” inputs; (ii) inputs that are uncorrelated from trial to trial, which we call the background noise. In consequence, the total synaptic input 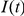 can be written as
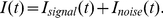
(25)


We further assume that both signal and noise inputs consists of a sum of large number of synaptic inputs, each individual synaptic input being of small amplitude. We therefore use the diffusion approximation [30], and represent both signal and noise inputs as Gaussian random processes. Within the diffusion approximation, the difference between a conductance-based input and a current-based input is merely a rescaling of the membrane time constant [68], hence here we consider only a current-based input.

For convenience, the mean of the input signal 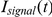 is taken to be zero. The correlation time 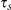 and the standard deviation 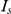 of 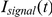 are parameters which we systematically vary in this study. Realizations of 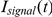 are generated using
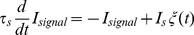
(26)where 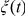 is a Gaussian process, with zero mean, unit variance and vanishing correlation time. The same realization of 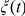 is used in all trials.

The background noise 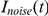 is a Gaussian process of mean 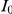, standard deviation 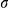 and vanishing correlation time, uncorrelated from trial to trial. The parameters 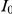 and 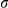 were systematically varied.

### Transfer function and linear response of integrate-and-fire neurons

Here we provide the summary of definitions and expressions for the transfer function and linear response functions of integrate-and-fire neurons. For completeness full derivations are provided below.

The transfer function 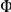 determines the average firing rate of a neuron in response to a steady input of the form 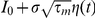, where 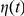 is a random process of zero mean and unit variance representing background noise, and 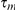 is the membrane time constant.

For the leaky integrate-and-fire neuron receiving background noise uncorrelated in time, the transfer function is given by [27]

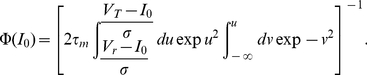
(27)


For the exponential integrate-and-fire neuron receiving background noise uncorrelated in time, the transfer function can be expressed as [21]

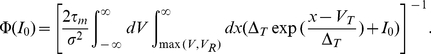
(28)A convenient method of evaluating Eq. (28) is to integrate the steady state Fokker-Planck equation [26].

The rate response function 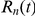 specifies the trial-averaged firing rate of the neuron in response to a time-varying input of small amplitude [12]. More precisely, in response to an input of the form 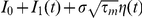, with 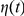 a Gaussian random process independent from trial to trial and 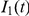 identical in all trials, at the linear order the firing rate of the neuron is given by
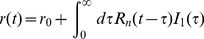
(29)where 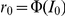. Taking the Fourier transform, Eq. (29) becomes
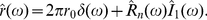
(30)where 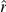, 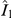 and 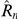 are the Fourier transforms of 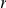, 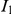 and 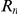.

For the LIF receiving a background noise uncorrelated in time, the response function in frequency 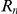 can be calculated from the Fokker-Planck equation [12], [24]. The result reads:
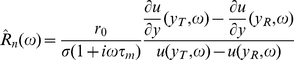
(31)where 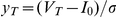, 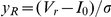, and 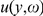 is given in terms of a combination of hypergeometric functions, or equivalently as the solution of the differential equation
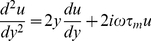
(32)with the condition that 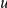 is bounded as 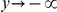.

For the EIF, no explicit expression is available for 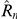, but a method has been developed for computing 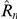 numerically from the Fokker-Planck equation [26]. That procedure is essentially equivalent to integrating Eq. (32) for the LIF model. This is the method we use here. For details, see [26].

### Comparison between model predictions and simulations

To assess the precision of the firing rates predicted by various models, we have systematically compared the predicted firing rates with results of simulations of the LIF, EIF and Wang-Buzsáki neurons.

The membrane potential dynamics of the neuronal models were simulated using a standard second-order Runge-Kutta algorithm with a time step of 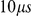. For each set of parameters, a fixed, 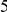 s long random instance of the input signal was applied in 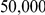 trials. The output firing rate was then computed by averaging over trials the spike trains binned in windows of 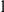 ms.

To obtain the predicted firing rates, the original input signal was sampled at intervals of 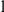 ms, convolved with the linear filter (determined with a precision of 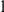 ms) and/or fed through the static non-linearity.

To compare quantitatively the prediction with the numerical firing rate, we computed the Pearson's correlation coefficient:
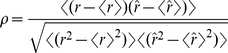
(33)where 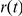 is the numerical firing rate and 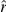 is the firing rate predicted by the model. The average is taken over time bins
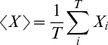
(34)where 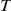 is the total number of bins.

The value of the Pearson correlation coefficient 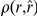, which lies between −1 and 1, represents the fraction of variance of 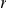 accounted for by a linear, instantaneous transformation of 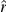. A caveat of the correlation coefficient is that its value can be high even if the means and variances of 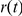 and 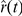 are very different: 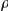 quantifies only the match between the temporal variations of the two time series. We have therefore checked separately that, when values of 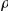 are high, the LN and rate models provide accurate predictions of the mean and variance of the firing rate (data not shown).

An alternative standard measure of the similarity between 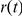 and 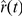 is the root mean square distance 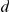 defined by
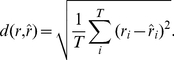
(35)


If the means and variances of the two time series are identical, there is a simple relationship between 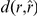 and 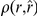:
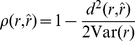
(36)


An advantage of the RMS distance 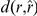 over the correlation coefficient 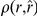 is that 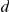 takes into account the match between the means and variances of 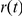 and 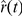. A disadvantage is that the scale of 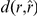 varies when the input parameters are varied. To compare the predictions of the models as the parameters are varied, we have therefore chosen the dimensionless measure provided by the correlation coefficient 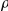.

For a fixed set of parameters, the RMS distance 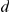 is a very useful comparison between the PSTH and various models, as it provides the mean error in units of Hz: we therefore display these values to the graphs comparing the predictions of different models for fixed parameter values (Figs. 3 A, 6 and 7).

The value of 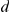 is bounded from below by the error induced by the finite number of trials used to estimate the PSTH. For a given instantaneous firing rate 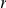, the error can be estimated to be 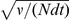 where 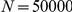 is the number of trials and 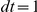 ms is the size of the time bin. As the instantaneous firing rates vary from 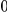 to 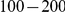 Hz in Figs. 3 A, 6 and 7, the error on the PSTH is of the order of 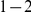 Hz. Note that this precision is far superior to the one that can be reached in experiments, where the number of trials is typically smaller by several orders of magnitude.

### Calculating the rate response function of the leaky integrate-and-fire neuron

For the leaky integrate-and-fire neuron receiving background noise uncorrelated in time, the rate response in frequency 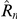 can be obtained from the first-order perturbation of the steady-state Fokker-Planck equation [24]. The original perturbation study [24] was done in the context of a recurrent inhibitory network, but the rate response response function can be deduced in exactly the same way [12], [25]. Here we provide the direct derivation of the rate response response function, following the same steps as in [24].

We consider a leaky integrate-and-fire neuron with membrane potential dynamics defined by Eq. (17), receiving an input current of the form
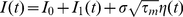
(37)where 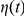 is a Gaussian white noise process of zero mean and unit variance, uncorrelated from trial to trial.

To study the stochastic dynamics of the membrane potential, we look at the probability distribution of the membrane potential 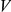 as function of time. The dynamics of the corresponding probability density 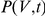 obey the Fokker-Planck equation [69]:
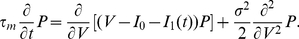
(38)


This equation expresses the conservation of probability in time, and can also be written as
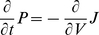
(39)where the current of probability density 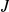 is given by
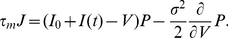
(40)


The instantaneous firing rate is given by the flux of probability density through the threshold membrane potential 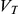:
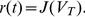
(41)


The membrane potential is reset to 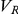 after crossing the threshold, hence 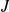 is discontinuous at 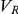 and obeys:
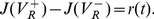
(42)


As the membrane potential cannot exceed the threshold, for 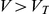 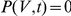. Since the probability density current 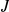 depends on the derivative of 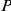 with respect to 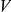, 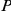 must be a continuous function of 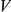, hence
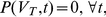
(43)

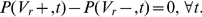
(44)


Eqs. (41–44) are the four boundary conditions for the Fokker-Planck Equation. In addition we will require that 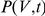 be integrable as 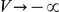.

#### Steady state

If 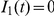, the neuron receives a steady input current, and its output rate is constant in time, 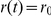. Eqs. (41) and (42), then imply that 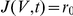 for 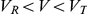 and 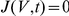 for 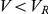. From Eq. (40) the steady-state probability density 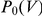 is proportional to 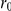. Integrating Eq. (40) determines 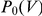 up to a multiplicative constant, which is obtained from the normalization condition for 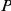. The final result reads
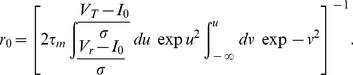
(45)


#### First order perturbation

For convenience, we now introduce the rescaled notations:
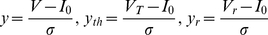
(46)

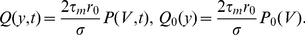
(47)


To calculate the linear perturbation of the firing rate arising from a time-varying input current 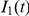 we write
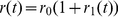
(48)

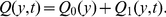
(49)


Keeping only first-order terms, the Fokker-Planck equation becomes
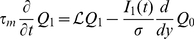
(50)where 
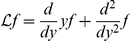
, and the boundary conditions read
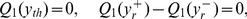
(51)

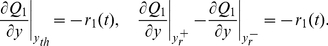
(52)


To solve Eq. (50), we take its Fourier transform which yields
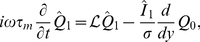
(53)where 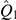 and 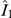 are the Fourier transforms of 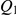 and 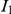.

In Fourier space, the boundary conditions become
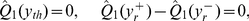
(54)

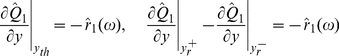
(55)where 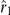 is the Fourier transform of 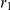.

The solution of Eq. (53) can be expressed as
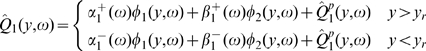
(56)where 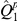 is a particular solution of the non-homogeneous equation and 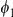 and 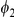 are two independent solutions of the homogeneous equation.

As shown in [24], a particular solution is given by
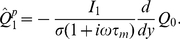
(57)


The homogeneous equation reads
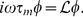
(58)


Two independent solutions of Eq. (58) can be expressed as [24]

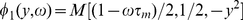
(59)

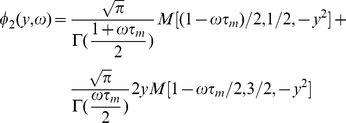
(60)where 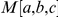 are confluent hypergeometric functions [70].

The full solution for 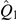 is obtained by determining the four unknown coefficients 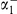, 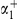, 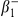 and 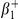 from the four boundary conditions. The boundary conditions however depend on 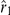, which is not known at this point.

This discrepancy is resolved by noting that 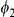 decays exponentially as 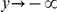 while 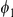 decays algebraically. Hence only 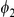 is integrable, and if 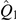 is to be integrable we must have 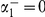. Enforcing this condition leads to
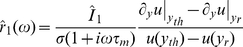
(61)where
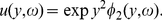
(62)


In conclusion, we have
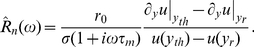
(63)


Note that the function 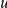 is a solution of
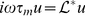
(64)where 
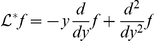
, i.e. 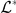 is the operator adjoint to 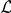. In practice, when evaluating Eq. (61), it is often preferable to evaluate 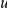 by integrating Eq. (64) rather than using available implementations of the confluent hyper-geometric functions.

#### Limit of vanishing firing rate

We consider here the limit 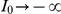, keeping 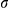 fixed. In that limit 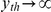 and 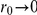. In the limit 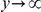, we have ( [70], Eq.13.5.1)
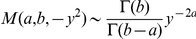
and therefore
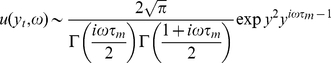
leading to
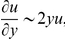
and therefore
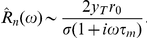
The next step is to relate the numerator of the above equation to the derivative of 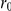 with respect to the mean input. Starting from the equation for the f-I curve, 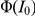, we find that
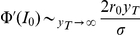
and therefore
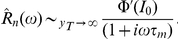
so that in the limit of 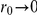 the rate response function of the LIF model becomes proportional to the impedance of the voltage. Note that the limits 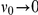 and 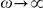 do not commute.
